# Unveiling Learning Strategies in the Mirror-Drawing Task: A Single-Case Study of Movement Stability and Complexity Using Entropy

**DOI:** 10.3390/e27050484

**Published:** 2025-04-30

**Authors:** Hiroki Murakami, Norimasa Yamada

**Affiliations:** 1 Faculty of Interdisciplinary Economics Department of Interdisciplinary Economics, Kinjo University, 1200 Kasama-machi, Hakusan 924-8511, Ishikawa, Japan; 2 Graduate School of Health and Sport Sciences, Chukyo University, 101 Tokodachi, Kaizu-cho, Toyota 470-0393, Aichi, Japan

**Keywords:** mirror-drawing test, motor learning, entropy analysis, movement stability, control strategy adaptation

## Abstract

The mirror-drawing task has been widely used in motor learning research to investigate procedural memory and movement control. However, studies have primarily focused on global performance measures such as movement time and the number of errors and lack insight into localized learning patterns. This case study aimed to analyze motor learning characteristics by combining traditional measures with entropy analysis, a method for capturing movement stability and complexity. Using a star-shaped figure divided into 12 segments, a single participant performed 100 trials of the mirror-drawing task. The movement coordinates were recorded at 60 Hz using a stylus on a mirrored tablet screen. The results showed that movement time decreased over the trials and entropy values showed an initial increase, followed by a decrease, suggesting exploratory behavior and subsequent stabilization. In particular, the interference side segments requiring complex visual–motor transformations showed prolonged instability and delayed control stabilization compared with the noninterference side segments. The integration of entropy analysis allowed a clearer visualization of the trial-and-error phases and movement instability, providing novel insights into the motor learning process. These findings, though limited to a single case, contribute to the understanding of adaptive movement control strategies and suggest that local learning properties should be considered in skill acquisition research.

## 1. Introduction

The mirror-drawing task is a psychological and neuroscientific task that is widely used to study learning, motor control, and brain function. In the 1890s, William James [1] referred to habit formation in his book “*Principles of Psychology*” and suggested that this concept of habit could be the basis for the measures of procedural memory and learning used in later psychological research. From the 1910s to 1920s, the concept was widely used in the field of learning and skill acquisition, and, in the 1930s, Snoddy [2] studied the learning curve using this task and demonstrated the “law of practice” in the acquisition of motor skills. Furthermore, in Milner’s [3] study of patients with anterograde amnesia, the mirror-image depiction task attracted attention as an important method for clarifying the separation of procedural and episodic memory. More recently, studies using functional magnetic resonance imaging and transcranial magnetic stimulation have shown that brain regions such as the cerebellum, basal ganglia, and prefrontal cortex play an important role during the mirror-image task [4] and are crucial in research on movement learning and cognitive function.

The learning process of mirror-image display tasks has often been studied using movement time and the number of errors as indices. Although this has allowed us to capture the learning process in the task as a whole, a detailed analysis of which parts of the task were error-prone and in which sections learning stagnated was not performed sufficiently. To address this issue, Hotta et al. [5] used a method to characterize motion and learning progression in specific sections by capturing the motion of the stylus tip during a mirror-image drawing task using a 300 Hz high-speed camera and dividing the figure into several sections. The results showed that not only were the total movement time and number of deviations reduced, but there were also intervals in which learning progressed and intervals in which learning stagnated. Although this study suggests that examining the local characteristics of movement in the learning process of mirror-image display tasks is important, the limited amount of data and improved trajectory analysis methods require a more detailed analysis.

Recently, entropy analysis has been introduced to further understand the complexity of motion trajectories. Information entropy, proposed by Shannon [6], quantifies the uncertainty and randomness of data and is considered useful for capturing movement instability and complexity in movement control and learning processes [7]. Entropy analysis has been applied to motor learning and movement control research from the perspective of the dynamic systems theory and nonlinear dynamics to assess the complexity and unpredictability of time-series data [8,9,10]. In particular, sample and approximate entropy are suitable for assessing the regularity or randomness of variation patterns of movement trajectories [11], whereas multiscale entropy has attracted attention as an analytical method for capturing short- and long-term variations in movement control [12]. Murakami and Yamada [13,14] conducted an entropy analysis of hand movement trajectories and showed that movement flexibility and adaptive changes were important for performance improvement. These findings support the notion that entropy analysis is a useful indicator of movement learning. Therefore, applying these entropy measures to a mirroring task is expected to reveal further details regarding the degree of movement flexibility and adaptation during the learning process [15]. Conventional assessments based solely on the number of errors or movement times only provide superficial performance indicators. By contrast, an analysis of the number of movement trajectory deviations and deviation points allows a detailed examination of which parts of the trajectory are stable and prone to errors [16]. Furthermore, entropy analysis helps us to identify and quantify random movements in the early stages of learning, thereby providing insights into the development of more efficient movement patterns [17]. This method captures qualitative changes in movement learning and strategy and provides a deeper understanding of the learning process. These findings support the notion that entropy analysis is a useful indicator of movement learning.

In addition to our previous work, recent studies by other research groups have also highlighted the growing relevance of entropy and other nonlinear analysis methods in motor learning and rehabilitation. For example, Asghari et al. [8] demonstrated that multiscale entropy can sensitively detect age-related differences in movement variability. Park and Han [9] compared linear and nonlinear models and confirmed the utility of nonlinear approaches for modeling complex adaptive motor behaviors. Smith et al. [18] used approximate entropy to assess spontaneous leg movements in infants with myelomeningocele and demonstrated that reduced complexity may reflect impaired neuromotor control. Similarly, Deffeyes et al. [19] employed approximate entropy to evaluate postural sway in infants with developmental delays, suggesting its usefulness for identifying instability in the development of sitting control. In Japan, Tomita [20] discussed the role of motor variability in rehabilitation and highlighted the value of nonlinear indices such as recurrence quantification analysis and sample entropy for understanding sensorimotor adaptation. Similarly, Kodama [21] explored the application of nonlinear time-series analysis to biological signals and argued for its potential in assessing subtle temporal dynamics in motor control and rehabilitation contexts. These studies strongly support the broader applicability of entropy-based and nonlinear analytical approaches, reinforcing the importance of our methodology in this expanding area of research.

Therefore, in this study, in addition to the conventional indicators of movement time and number of deviations, we introduced movement speed and entropy analysis to investigate the detailed process of motor learning in the mirror-drawing task and examined changes in control strategies and fluctuations in movement stability during the learning process. We created a mirror-drawing task on a PC using the psychological experimentation software PsychoPy, installed a screen that mirrored the tablet on the mirror-drawing device, and recorded the coordinates of the stylus tip that drew on the screen at 60 Hz. The star-shaped figure used in the task was divided into 12 segments, which were classified into interference (involving oblique movements under depth-reversed feedback and requiring angular remapping) and noninterference (primarily involving linear front-to-back movements with simpler directional transformation) segments. Through this analysis, this study aimed to clarify which parts of the learning process involve trial and error and the establishment of stable control strategies.

## 2. Materials and Methods

### 2.1. Participant

A healthy, right-handed man (20 years old) enrolled at the researchers’ university participated in this study. The participant self-reported having normal vision and no motor impairments. Although only one participant was included, this study employed an intensive design with 100 repeated trials. This allowed for the detailed observation of gradual changes in movement control and learning. This study was designed as a pilot investigation to explore the feasibility of using entropy analysis to capture the localized features of motor learning. All human study procedures were conducted in accordance with the Declaration of Helsinki and the ethics code of Chukyo University and were approved by the ethics committee of Chukyo University on 26 February 2024 (approval number: 2023–089). The participant provided oral informed consent prior to participation.

### 2.2. Apparatus

**Figure 1:**
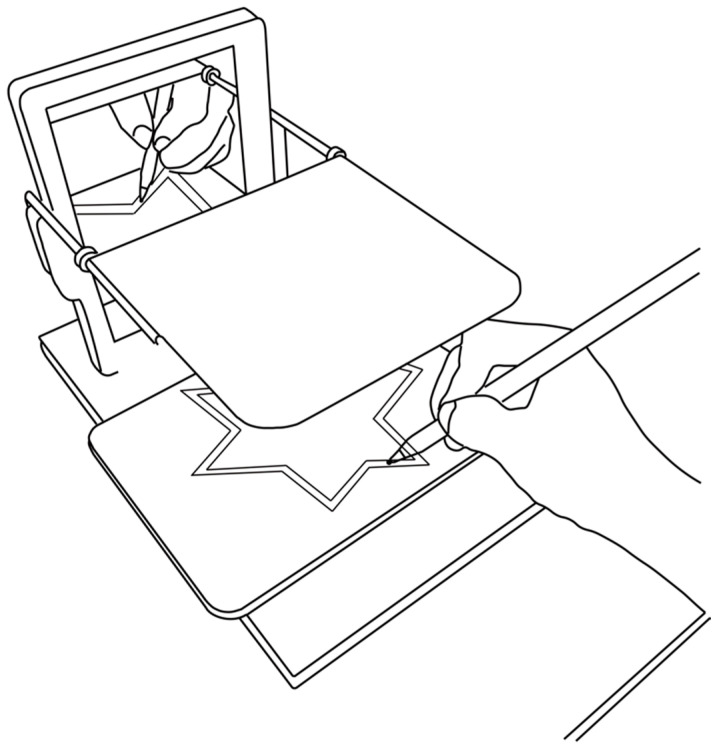
Conceptual diagram of the experimental setup for the mirror-drawing task using the PsychoPy system. The participants viewed a star-shaped figure mirrored on a tablet screen and traced the inside using a stylus. The figure was divided into 12 segments.

We created a mirror-drawing task using the psychological experiment software PsychoPy (ver. 2023.2.3). The task involved a star-shaped figure composed of 12 sides of equal width and length (length: 210 pixels; width: 20 pixels; actual size on the tablet: length 1.25 cm, width 0.12 cm). A tablet (iPad mini 6th generation, Apple, Infinite Loop Cupertino, CA, USA) was attached to the mirrored drawing device, and the task created on a PC (MacBook Pro 14 inch, Apple) was mirrored. The 2D coordinates of the stylus tip were obtained at 60 Hz by tracing the screen on the tablet with a stylus (Apple Pencil 2nd Generation, Apple) while looking at the stylus and the screen reflected in the mirror (Figure 1). The cursor size of the stylus tip displayed on the tablet was a circle with a width of 10 pixels (actual size: 0.06 cm).

### 2.3. Experimental Design

**Figure 2:**
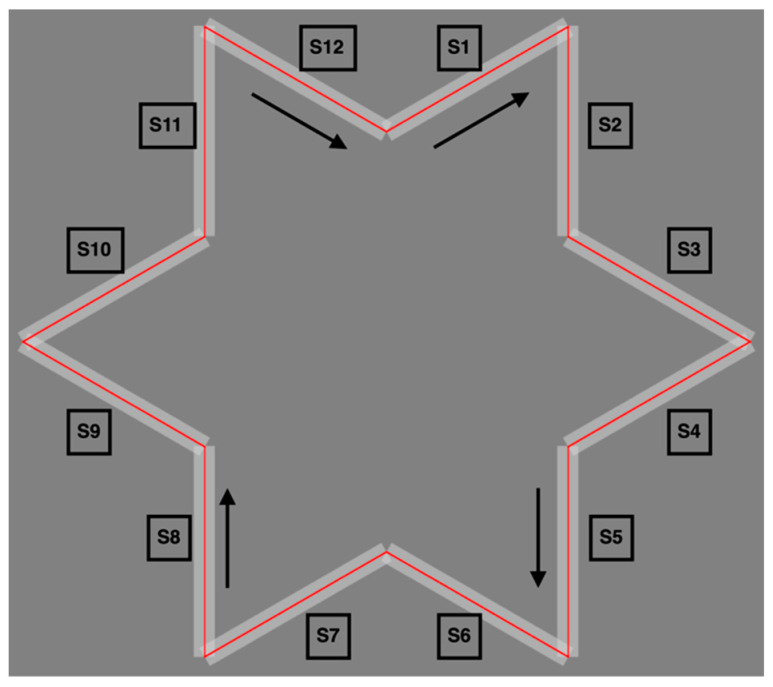
Structure of the star-shaped figure and segment classification. A star-shaped figure was used in the mirror-drawing task. The 12 sides of the segment were categorized into interference (S1, S3, S4, S6, S7, S9, S10, and S12) and noninterference (S2, S5, S8, and S11) segments, which were used to analyze the differences in motor learning strategies.

The participant was instructed to trace the star-shaped figure shown in Figure 2 in a clockwise direction (S1–S12) while looking at the screen with a stylus in their dominant hand, which was reflected in a mirror. In doing so, he was asked to perform the following: (1) trace as quickly as possible without going outside of the frame as much as possible; and (2) if it was judged that he had gone outside the frame, the cursor of the stylus displayed on the screen would change from black to red, indicating that he was to return to the frame immediately. The participant was not given any instructions regarding their body positions, and they only looked at the screen. They were able to self-report and were allowed to rest sufficiently during the test. In addition, the participant was asked to self-report whether they needed additional rest during the test to ensure minimal fatigue. Errors were operationally defined as instances in which the recorded coordinates of the stylus tip deviated from the boundary of the star-shaped figure. The frame of the figure was mathematically defined as a 20-pixel wide band along each side segment. During each trial, all stylus tip coordinates (sampled at 60 Hz) were compared with the frame boundaries, and the deviations were logged as error points. The total number of errors per trial and the spatial coordinates of each error were extracted for analysis.

### 2.4. Experimental Procedure

The participant entered the laboratory, sat in a chair in front of a desk containing the experimental equipment, and was given an explanation of the task and risks of the experiment. They chose to perform the task with their dominant right hand. Before the experiment, the participant was able to get a feel for the stylus on the tablet without the use of a mirror. As the experiment was set up to record the time when the tip of the stylus touched the start-position area, the experimenter held the participant’s hand and moved it to the start position, and the participant grounded the stylus on the tablet at their own time to start the trial. A total of 100 trials were conducted, and the participant did not receive any feedback on their performance until all trials were completed.

### 2.5. Data Analysis

All numerical calculations, including the analyses, were performed using Mathematica 12.3.1.0 (Wolfram Research, Champaign, IL, USA).

#### 2.5.1. Calculation of Movement Time Across Trials and Segments

Using the 2D coordinates obtained at 60 Hz, the following two types of movement times (MT[s]) were calculated: (1) the time taken for the entire trial from start to finish (MT_all_) and (2) the time taken for each side of the figure (MT_sidenum_, e.g., MT_1_ for side 1).

#### 2.5.2. Detection of Peak Velocity and Its Temporal Variability

The velocity was calculated using the first-order derivative of the time-series coordinates of each axis. In addition, both were smoothed with a bilateral fourth-order Butterworth filter (5 Hz low-pass filter) and synthesized using the values of each axis. To identify the rapid changes required for movement correction, the number and magnitude of peak velocities in each trial were calculated using the mean × 1.5 standard deviation (SD) of each trial as the threshold.

#### 2.5.3. Quantifying Stability and Complexity Using Distance and Entropy

As mentioned in the introduction, Shannon’s [6] information entropy has recently gained attention in the field of human movement analysis and has been widely used as an indicator to evaluate the randomness and stability of movement [7]. Entropy is a measure that quantifies the difficulty of predicting a system and its uncertainty and is an effective indicator of irregularity in movement and the complexity of control in the process of movement control and learning [13,14,22].

**Figure 3:**
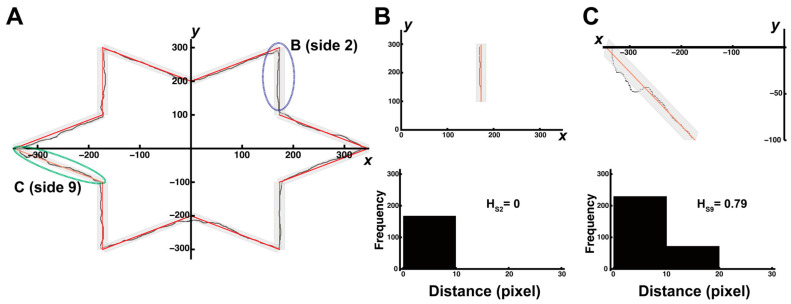
(**A**) Representative trajectory plots and entropy values during the early stage for Segment (**B**) (Side 2; noninterference) and Segment (**C**) (Side 9; interference). Segment (**C**) shows dispersed and less stable movements with a higher entropy value (e.g., 0.79), whereas Segment (**B**) shows tightly clustered trajectories with an entropy of 0. These comparisons illustrate that entropy analysis effectively captures differences in movement stability between segments, even when visual inspection may be ambiguous.

Figure 3 presents a representative example of the stylus trajectories in a mirror-drawing task. These examples include interference (Segment B) and noninterference (Segment C) segments, which inherently differ in their complexity of visual–motor transformations. In this study, we incorporated the concept of entropy to examine the changes in the stability of the depicted trajectory and the transition in the complexity of movement control as the trial (learning) progressed. Entropy analysis is expected to play an important role in analyzing how movement stability changes as trials progress under conditions where visual–motor transformation must be replanned, such as in mirror-drawing tasks.

The shortest distance from the central straight line (red line in Figure 2 and Figure 3) within each 12-sided frame was calculated for each coordinate. Based on the distance values, entropy was calculated using the method described in previous studies [13,14,22]. The width of bin i used in this study was set to 10 pixels, which was the deviation from the center line within the frame. This entropy was calculated using $H_{1}(X)\equiv \underset{a\to 1}{\lim}H_{a}\left(X\right)=\sum P_{i}\log_{2}\left(1/P_{i}\right)$, where $P_{i}$ was the frequency distribution of data points in bin i. The limiting value of $H_{a}$ as a→1 was the Shannon entropy [6,23]. Therefore, if the distance from the shape frame remained within the same bin throughout the trial, the entropy was 0. Specifically, if the distance of all coordinates was within 10 pixels, this indicated no errors from inside the star-shaped figure throughout the trial. In this case, the entropy was 0. Figure 3B,C show the actual trajectories and corresponding histograms of the shortest distances from the red centerline (bin width = 10 pixels) for Segments B (S2) and C (S9), respectively. The entropy values calculated from the histogram are shown in Figure 3. The visual differences in trajectory patterns were aligned closely with the calculated entropy values. This reinforces the validity of entropy as an indicator of movement complexity and stability.

The integration of visual and quantitative data underscores the strengths of this study’s methodology. This combined presentation highlights how entropy analysis can effectively visualize and quantify movement variability and complexity. This approach reinforces the reliability and interpretability of the results and provides a comprehensive understanding of how movement stability evolves during motor learning.

### 2.6. Statistical Analysis

Each variable was calculated by classifying it into the following three categories: the entire trial (all from L1 to L12), interfering sides (S1, S3, S4, S6, S7, S9, S10, and S12), and noninterfering sides (S2, S5, S8, and S11). Because of the possibility of the data for each trial varying greatly for all variables (MT, velocity, distance, and entropy), we divided the data into 10 blocks (e.g., Block 1: 1–10 trials, Block 2: 11–20 trials…), calculated the average value for each block, and analyzed the data trends. We also performed regression analysis on the values for each block and statistically examined the changes that occurred as learning progressed.

## 3. Results

### 3.1. Learning-Related Changes in Movement Time Across Trial Blocks

**Figure 4:**
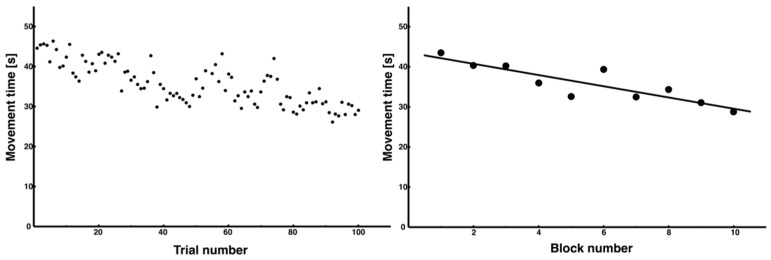
Changes in the movement time across all trials. The movement time for each trial is also presented. Although it was difficult to discern trends from the data of all 100 trials (**left**), which showed considerable variability in individual trials, the decreasing trend when the average of 10 trials was used as 1 block indicated that the efficiency of the movement performance was improving (**right**).

The average MT for all 100 trials (MT_all_) showed a high degree of variability between trials, making it difficult to confirm a consistent trend as learning progressed (Figure 4, left). However, when the average block time was calculated for each block of 10 trials, a trend of decreasing movement time with an increasing number of trials was confirmed (Figure 4, right). This trend showed a significant negative slope in the regression analysis of a linear function (regression equation: y = –1.40x + 43.53, *R²* = 0.75).

**Figure 5:**
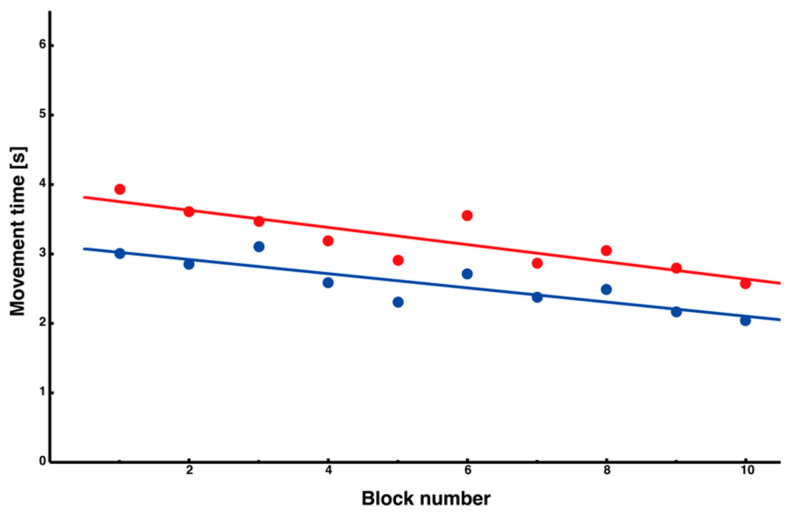
Changes in the movement time of each side segment. The movement time for each side segment is presented (interference side: red; noninterference side: blue). A negative correlation, which tended to decrease as learning progressed, was observed in both segments using linear regression analysis.

Subsequently, we analyzed the interference and noninterference sides using only the block average data and found that, in all conditions, the movement time tended to shorten as the trial progressed. A significant negative slope was observed in the linear regression analysis (Figure 5, interference (red): y = –0.12x + 3.88, *R*^2^ = 0.72; noninterference (blue): y = –1.10x + 3.12, *R*^2^ = 0.73). Furthermore, the noninterference side consistently had shorter movement times from the start of the trial than the interference side, and there was no evidence that the movement time of the interference side approached that of the noninterference side. These findings suggest that learning oblique movements with depth-reversed visual feedback depends on complex information processing and motor planning.

### 3.2. Adaptive Changes in Peak Velocity and Motor Strategy

**Figure 6:**
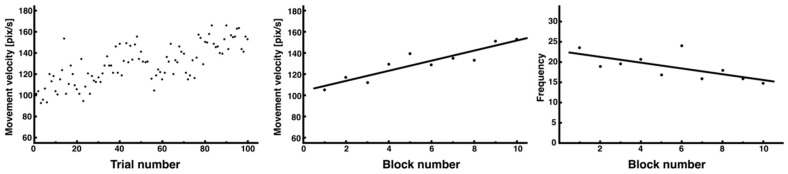
Changes in performance variables (peak velocity and number of times above threshold) related to the movement velocity analysis. The average peak velocity for each trial is presented. Although it was difficult to discern trends from the data of all 100 trials (**left**), which showed considerable variability in individual trials, an increasing trend was observed when the average of 10 trials was used as 1 block (**middle**). In addition, the average number of peak velocities, with 10 trials as 1 block, showed a decreasing trend as learning progressed (**right**).

In the dataset of all 100 trials, the variation in the peak velocity was large, making it difficult to visually confirm consistent trends (Figure 6, left). After calculating the block average for each block of 10 trials, the peak velocity increased as the trials progressed (Figure 6, middle), and there was a trend toward a decrease in the rapid fluctuations in velocity (the number of times the threshold was exceeded) (Figure 6, right). The regression analysis showed a significant linear relationship for all variables (peak velocity: y = 4.77x + 104.11, *R*^2^ = 0.82; frequency: y = –0.71x + 22.71, *R*^2^ = 0.38).

**Figure 7:**
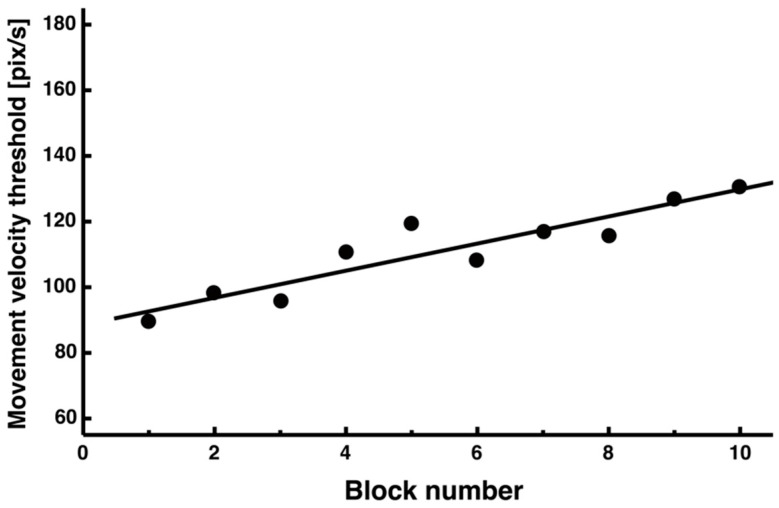
Changes in threshold block average (1 block of 10 trials) when analyzing peak velocity.

Furthermore, the average value of the threshold (calculated from the average of each trial × 1.5 SD) for calculating the peak velocity of movement also tended to increase as the trials progressed (Figure 7, y = 4.13x + 88.52, *R*^2^ = 0.84).

**Figure 8:**
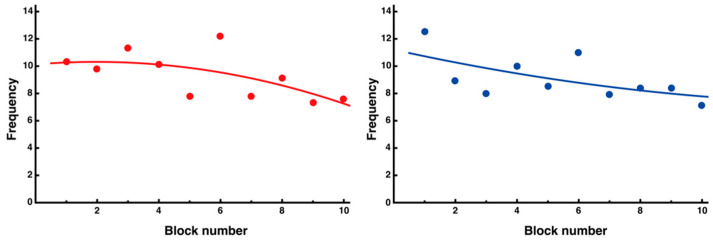
Changes in number of peak velocities for each side segment. The number of peak velocities for each side segment is presented (**left**: interference side (red); **right**: noninterference side (blue)). Negative correlations, which tended to decrease as learning progressed, were observed in both segments using nonlinear regression analysis; however, the noninterference side showed a decrease from the beginning of learning. The quadratic functions fitted to each data point are also shown.

The average block number of peak velocities for the noninterference side showed a relatively stable trend from the beginning of the trials (Figure 8, right). Although the regression analysis showed a slightly decreasing trend, the fluctuation in the values was small, and it was assumed that a certain control strategy was established from the beginning of learning (y = 0.02x^2^ − 0.50x + 11.21, *R*^2^ = 0.37). By contrast, the average block number of peak velocities on the interference side per 10 trials showed an exploratory change, with a transient increase from the beginning to the middle of the trial, followed by a decrease (Figure 8, left). The regression analysis showed a nonlinear change from the initial increase to the final decrease, indicating the presence of trial and error in the learning process (y = −0.05x^2^ − 0.19x + 10.13, *R*^2^ = 0.41).

### 3.3. Trial-and-Error Patterns Captured by Distance and Entropy Analysis

**Figure 9:**
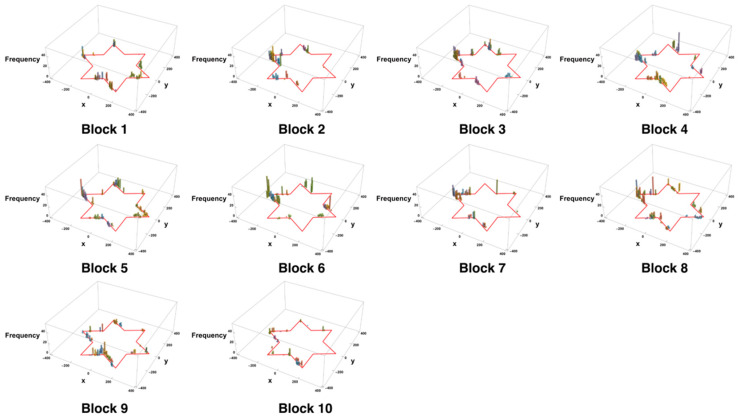
Histogram of error occurrence coordinates in each block (10 trials per block). Bin size is 10 pixels. The height of the bar is indicative of the frequency of errors, and the color of the bar changes in accordance with the height setting designated by Mathematica s Histogram3D default parameters.

When the histogram of the coordinates of errors that occurred was shown in blocks of 10 trials (Figure 9), a qualitative analysis confirmed that the errors tended to be concentrated near the switching points between the interfering edges (e.g., between S6 and S7 and between S9 and S10). This result suggests that switching between movement plans and spatial replanning might have contributed to the errors.

**Figure 10:**
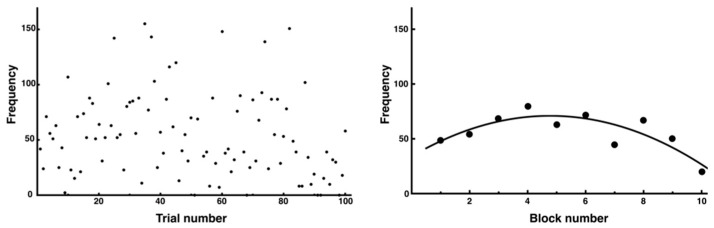
Changes in number of errors across all trials. The number of errors in each trial is presented. Although it was difficult to discern trends from the data of all 100 trials (**left**), which showed considerable variability in individual trials, an inverted U-shaped trend was observed when the average of 10 trials was used as 1 block (**right**).

In addition, the number of errors in the data from all 100 trials varied widely, making it difficult to confirm consistent trends (Figure 10, left). However, when the block average was calculated for each block of 10 trials, a tendency was confirmed where the number of errors temporarily increased at the beginning of the trial and then decreased (Figure 10, right). The regression analysis revealed a significant quadratic relationship (y = −1.62x^2^ + 15.49x + 34.01, *R*^2^ = 0.71). This is thought to reflect the process of gradual stabilization after an increase in trial and error in movement learning.

**Figure 11:**
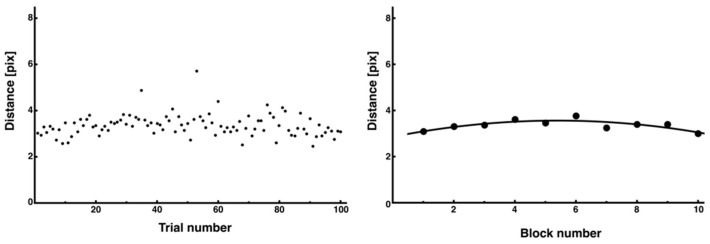
Changes in average distance across all trials. The average distance for each trial is also presented. Although it was difficult to discern trends from the data of all 100 trials (**left**), which showed considerable variability in individual trials, an inverted U-shaped trend was observed when the average of 10 trials was used as 1 block (**right**).

The average distance for all 100 trials showed a high degree of variability between trials, making it difficult to confirm a consistent trend as learning progressed (Figure 11, left). However, when the average block time was calculated for each block of 10 trials, there was a trend of a transient increase at the beginning of the trial, followed by a decrease (Figure 11, right). This trend was significant in the regression analysis of a nonlinear function (y = −0.02x^2^ + 0.26x + 2.86, *R*^2^ = 0.67).

**Figure 12:**
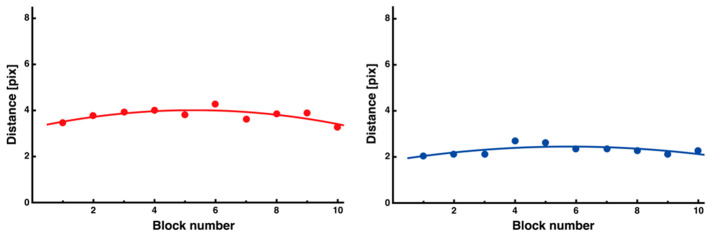
Changes in average distance for each side segment. Both side segments showed an inverted U-shaped trend, with smaller values on the noninterfering side (**right**) than on the interfering side (**left**). The quadratic functions fitted to each data point are also shown.

After calculating the average block size for each of the 10 trials, it was confirmed that the distance on both sides temporarily increased at the beginning of the trial and then decreased, and that the distance was consistently longer on the interfering side than on the noninterfering side (Figure 12, interference (red): y = −0.03x^2^ + 0.29x + 3.25, *R*^2^ = 0.56; noninterference (blue): y = −0.02x^2^ + 0.21x + 1.85, *R*^2^ = 0.45).

**Figure 13:**
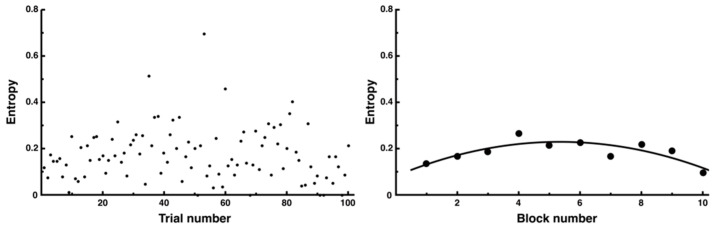
Changes in entropy across all trials. The entropy for each trial is presented. Although it was difficult to discern trends from the data of all 100 trials (**left**), which showed considerable variability in individual trials, an inverted U-shaped trend was observed when the average of 10 trials was used as 1 block (**right**).

The entropy calculated from the distance showed a large amount of fluctuation in the data from all 100 trials on all sides, making it difficult to confirm consistent trends (Figure 13, left). Therefore, when examining the average of each block of 10 trials, a tendency was observed where, similar to distance, the entropy temporarily increased at the beginning of the trial and then decreased (Figure 13, right, y = −0.01x^2^ + 0.06x + 0.08, *R*^2^ = 0.68). In particular, the increase in entropy coincided with the increase in errors, and the high number of errors may reflect an increase in movement replanning and trial and error.

**Figure 14:**
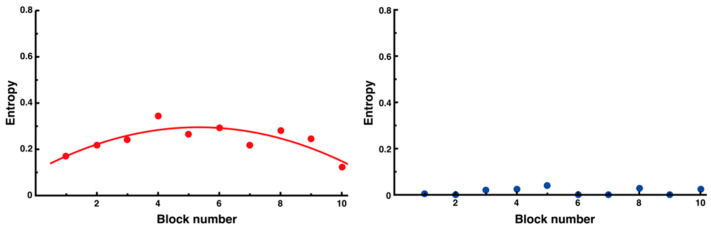
Changes in entropy for each side segment. The interfering side segments showed an inverted U-shaped trend (**left**), whereas the noninterfering side segments always showed a value of approximately 0 (**right**). The quadratic functions fitted to the data of the interfering side segments are also shown.

Furthermore, when the block average was calculated for every 10 trials, it was confirmed that the value of the noninterfering side was always approximately 0 (Figure 14, right), whereas the value of the interfering side tended to temporarily increase at the beginning of the trial and then decrease, similar to the results for all sides (Figure 14, left, y = −0.01x^2^ + 0.07x + 0.11, *R*^2^ = 0.68).

## 4. Discussion

### 4.1. Consistency with Research Results and Existing Research

This study aimed to clarify the characteristics of motor learning in mirror-drawing tasks and the changes in control strategies observed during this process. Specifically, we created a task in which a participant drew a star-shaped figure with 12 sides using the psychological experiment software PsychoPy. We then comprehensively analyzed the learning process of movement by acquiring coordinate data during the drawing. We used indices widely used in previous studies, such as movement time [24,25] and the number of deviations from the shape [26,27], as well as new indicators, such as movement velocity, the shortest distance from the line in the shape, and distance entropy. Subsequently, we examined the changes in movement stability and complexity in more detail. In addition, previous studies generally used the overall time from the start to the goal point of the figure and the number of deviations; however, this study calculated data for each side of the figure and focused on the characteristics of specific sides where learning progressed (or stalled) and the differences in learning between sides that required part movement and mirror-based depth inversion with angle changes (interfering sides) and sides that required only mirror-based depth inversion without angle changes (noninterfering sides). Through these investigations, we aimed to clarify how the differences in the progress and difficulty of learning are affected by the structure of the figures and physical constraints.

Although the trial-by-trial data exhibited high variability, likely reflecting exploratory movements and moment-to-moment corrections, blockwise averaging revealed clear learning trends in movement time, peak velocity, error frequency, and entropy. This supports previous findings [24,25] showing that motor learning involves random fluctuations and gradual stabilization, particularly in tasks that require novel visuomotor transformations.

In particular, the early stages of trials are expected to show a lot of exploratory movement, and many trials are required before an appropriate control strategy is established. The results of this study are consistent with this finding, confirming that trial-by-trial data are easily buried in noise and that block analysis is effective in revealing the essential trends of learning.

Furthermore, Serrien et al. [25] reported that there is a strong requirement for interhemispheric cooperation during mirror movements; that this cooperation is unstable, especially in the early stages of learning; and that this instability can lead to variations in movement performance. In this study, the marked variation in the 100-trial data for each variable might have been due to dynamic changes in the brain associated with movement replanning and adaptation.

### 4.2. Changes in Movement Velocity and Optimal Control Strategies

First, the threshold value for peak speed was calculated from the average speed of each trial × 1.5 SD, and this value increased as the second half of the trial progressed (Figure 7). The increase in the threshold value can be understood as the overall speed of drawing the figure increasing as the trial progresses, and this result can be considered consistent because the overall movement time was decreasing.

Second, the observed increase in peak velocity and simultaneous decrease in rapid velocity fluctuations suggest the acquisition of more efficient control strategies. This pattern is consistent with the optimal control theory [28], in which skilled performance reflects a balance between minimizing motor noise and maximizing efficiency. Notably, the delayed stabilization observed in the interference segments indicated that complex visual–motor transformations require prolonged reliance on feedback control before predictive (feedforward) control can emerge.

Furthermore, the fact that the stabilization of peak speed was delayed on the interference side compared with the noninterference side suggested that complex movements in mirroring require different information processing from normal movement control; moreover, there was a high degree of dependence on feedback control based on visual information. This is consistent with the suggestion that it takes time to revise the internal model and that a delay exists, particularly in situations where visual–motor conversion is complex [29].

### 4.3. Significance of Entropy Analysis in Evaluating the Stability and Complexity of Movements

In this study, to quantify the stability and complexity of movements, we introduced the distance from the center of the figure and entropy, in addition to the conventionally used indicators, and obtained new insights into the stability of movements and the complexity of control. Regarding the distribution of errors, we confirmed that the errors tended to be concentrated near the switching points on the sides (Figure 9). This is thought to reflect the fact that errors tend to occur in situations where movement must be replanned in tasks that involve spatial transformations, such as mirror drawing [25].

Entropy analysis provided a more nuanced understanding of motor learning than error counts alone. While the error frequency and entropy showed an initial increase followed by a gradual decrease, entropy captured the qualitative aspects of movement stability more clearly. In particular, the consistently higher entropy in the perturbed segments suggested greater instability and variability in control, likely due to the need for the ongoing recalibration of sensorimotor mappings. This finding supports the idea that learning in mirror tasks involves the resolution of perceptual–motor incongruence, especially when spatial transformations are the most complex.

### 4.4. Characteristics of Mirror Movements and Adaptation of Internal Models

As a result of the adaptation process specific to mirror movements, the results showed that the reduction in movement time and the stabilization of movement speed were delayed on the interfering side compared with the noninterfering side (Figure 5 and Figure 8). In particular, on the interfering side, instability persisted even in the second half of the trial, indicating that long-term adaptation was required compared with the noninterfering side. According to the internal model hypothesis [27], motor control is based on the prediction of the sensory consequences of movements. In the mirror-drawing task, especially in the interference segments, these predictions failed because of the mismatch between the intended motor actions and reversed visual feedback. The persistent difference in the movement time between the interference and noninterference segments, even after prolonged practice, suggests that the adaptation of the internal model remains incomplete or inherently limited in such complex conditions.

Furthermore, neuroimaging studies [25] have shown that mirror-related tasks activate brain regions such as the premotor cortex and parietal lobe areas associated with spatial reasoning and motor planning. These findings suggest that mirror learning may involve higher-order visuospatial processing beyond those involved in typical motor adaptation. Thus, successful learning may depend not only on motor repetition, but also on the reorganization of sensorimotor coordination at the cortical level, which requires further neuroscientific investigation.

### 4.5. Significance and Applicability

The scientific significance of this research lies in the fact that it analyzed the course of motor learning in a mirror-drawing task from a general trend and partial characteristic perspective. In previous research, the general approach was to focus on the movement time and number of deviations for the whole figure; however, in this research, by analyzing the movement data for each side segment of the figure, it was possible to identify the sections where learning stagnated and trial and error were concentrated. The novelty of this research is that it allowed us to gain a detailed understanding of the difficulties involved in mirror movements and situations in which it is necessary to reconstruct movement plans. In particular, the suggestion that learning mirror movements is not “learning as a whole” but involves “accumulating partial learning” is an important finding for rehabilitation and sports skills teaching. For example, the mirror therapy method used by Altschuler et al. [30] uses visual feedback to promote movement control. Our findings may also be applicable to the optimization of such methods (intensive intervention and design of areas where movement tends to stagnate).

However, this study has several limitations. First, although only one participant was included, it was designed as an in-depth exploratory pilot study with 100 consecutive trials. This intensive design allowed us to make fine-grained observations of the temporal dynamics of motor learning and segment-specific adaptation, which may not be easily captured in broader but less detailed designs.

Nevertheless, caution is required when generalizing these findings. First, individual differences in sensorimotor adaptation may be considerable, particularly under mirrored visual conditions. Replication with a larger and more diverse sample is required to evaluate the robustness and generalizability of the observed patterns. Second, this study did not include comparisons with other types of motor tasks, leaving the question of whether the findings are specific to mirror drawing unanswered. Third, the neurophysiological processes underlying internal model adaptation were not assessed, thus limiting insights into the brain-level mechanisms involved. Finally, while this study categorized figure segments as interference or noninterference segments based on the type of visuomotor transformation (specifically, whether the segment involved oblique movement under depth-reversed visual feedback), the actual difficulty experienced may exist along a continuum. For example, certain interference segments may require more directional switching or complex spatial remapping than others.

Future research should consider incorporating quantitative metrics such as the angular deviation between the hand and cursor movement vectors to more precisely assess the degree of visuomotor interference. This would provide a more nuanced understanding of local learning progression and sensorimotor adaptation.

### 4.6. Limitations and Future Directions

This study adopted an intensive single-case design involving 100 trials to investigate the segment-specific dynamics in mirror-drawing tasks using entropy analysis. This approach enabled a fine-grained, time-resolved analysis of motor adaptation under mirrored visual feedback and revealed detailed changes in stability and control strategies across specific trajectory segments. Although the findings of this study provide valuable insights, several limitations should be acknowledged.

First, although this study involved only one participant, the fundamental challenges posed by mirror-reversed drawing, such as disrupted visuomotor correspondence and spatial disorientation, are not unique to this individual but reflect universal features of human motor control. It is well-established that mirrored feedback inherently disturbs visuomotor mapping, and that task difficulty varies across movement directions owing to asymmetries in spatial remapping. Therefore, even with a single participant, the observed differences between the interference and noninterference segments possibly reflect generalizable properties of motor adaptation rather than idiosyncratic behavior. However, individual differences in visuomotor strategy and control flexibility remain important considerations. Future studies should include multiple participants in order to validate the consistency of segment-specific patterns in a broader population.

Second, although we categorized the figure segments as either interference or noninterference types, the complexity of visuomotor transformation possibly exists along a continuum. A more refined classification, incorporating angular deviation metrics, reaction latencies, or subjective difficulty ratings, may yield deeper insights into localized learning dynamics.

Third, although entropy analysis effectively captures the fluctuations in movement stability, it remains an indirect measure of cognitive or neural adaptation. The inclusion of physiological data, such as eye tracking, EMG, or neuroimaging, could enhance interpretability and provide a more integrated view of motor learning mechanisms.

In future research, this framework can be extended to other sensorimotor learning contexts, such as visuomotor rotation, gain adaptation, or inverted cursor control. These paradigms require spatial remapping and error-based adjustments, making them promising candidates for entropy-based, segment-level analyses.

## 5. Conclusions

In this study, we applied entropy analysis to a mirror-drawing task to examine the course of motor learning and control strategies. By analyzing both the overall trends and segment-level characteristics, we identified the differences in the learning process between the interference and noninterference segments. The results showed that the noninterference segments exhibited relatively stable control during the early stages of learning, whereas the interference segments were characterized by prolonged exploratory behaviors and delayed stabilization. The entropy analysis made it possible to visualize periods of instability and trial-and-error behaviors that are difficult to capture using conventional indicators. These findings suggest that mirror-image movements require continuous motor replanning and long-term adaptation, particularly in segments with high visuomotor conflict. This study highlights entropy as a valuable tool for assessing motor learning dynamics and identifying potential bottlenecks in skill acquisition. This contributes to a deeper understanding of the adaptive mechanisms specific to mirror-image movement tasks.

Although this study focused on mirror-drawing as a specific example of visuomotor transformation, its findings have broad implications. Generally, in motor learning, certain segments or phases of movement may progress more rapidly when appropriate sensory feedback is available, whereas others may stagnate due to feedback bias or ambiguity. The segment-specific differences observed here suggest that such localized variations in learning progression may be a fundamental property of motor adaptation, regardless of the task type or structure.

## Data Availability

The datasets and programming codes generated or analyzed in the current study are available from the corresponding author upon reasonable request.
